# A Roadmap for National Diabetic Retinopathy Screening in Croatia: Integrating European Evidence, Telemedicine, and AI

**DOI:** 10.3390/medicina62071251

**Published:** 2026-06-29

**Authors:** Toma Babić, Martina Tomić, Nenad Vukojević, Ivo Dumić-Čule, Sonja Jandroković, Tea Čaljkušić Mance

**Affiliations:** 1 Department of Diabetic Eye Complications, Vuk Vrhovac University Clinic for Diabetes, Endocrinology and Metabolic Diseases, Merkur University Hospital, 10000 Zagreb, Croatia; babicitoma@gmail.com (T.B.); martina.tomic@kb-merkur.hr (M.T.); 2 Department of Ophthalmology, Zagreb University Hospital Centre, 10000 Zagreb, Croatia; nvukojev@gmail.com (N.V.); sonja.jandrokovic@gmail.com (S.J.); 3 School of Medicine, University of Zagreb, 10000 Zagreb, Croatia; 4 Department of Nursing, University North, 104 Brigade 3, 42000 Varazdin, Croatia; 5 Department of Ophthalmology, Clinical Hospital Centre Rijeka, 51000 Rijeka, Croatia; teamance7@gmail.com; 6 School of Medicine, University of Rijeka, 51000 Rijeka, Croatia

**Keywords:** diabetic retinopathy, screening, telemedicine, artificial intelligence

## Abstract

Diabetic retinopathy (DR) is a leading cause of preventable blindness among working-age adults. Systematic screening programmes in the United Kingdom, Ireland, and the Nordic countries have reduced diabetes-related visual loss, yet many European countries, including Croatia, lack organised screening. This narrative review examines European DR screening programmes, evaluates telemedicine and artificial intelligence (AI) as enabling technologies, and proposes a phased roadmap for a national programme in Croatia. Croatia has nearly 400,000 registered persons with diabetes, a national diabetes registry (CroDiab) linked to the Central Health Information System (CEZIH), and pilot screening data showing 40% DR prevalence among screened patients with type 2 diabetes. The proposed programme combines decentralised telemedicine-based imaging at primary care sites with centralised grading, a staged rollout across three phases targeting 400,000 annual screenings, and stepwise AI integration for triage. Successful European programmes share standardised digital imaging, trained grading workforces, embedded quality assurance, and registry linkage. With dedicated funding and sustained political commitment, Croatia could adopt a hybrid telemedicine–AI model and, under favourable implementation conditions, reduce the burden of advanced DR over the coming decade.

## 1. Introduction

Diabetes mellitus (DM) now affects an estimated 537 million adults worldwide, a figure projected to reach 643 million by 2030 (the most recent IDF estimate available at the time of writing) [1]. Diabetic retinopathy (DR), the most common microvascular complication of DM, remains a leading cause of preventable blindness among working-age adults in high-income countries and an increasingly recognised burden in low- and middle-income settings [2,3]. The pathophysiology is well characterized: chronic hyperglycemia drives capillary damage that progresses through non-proliferative stages to proliferative disease and diabetic macular edema (DME). Decades of evidence from the Diabetic Retinopathy Study, the Early Treatment Diabetic Retinopathy Study (ETDRS), and the United Kingdom Prospective Diabetes Study (UKPDS) have established that timely detection and treatment can prevent the most severe visual loss [4,5,6].

Systematic screening programmes transform this biological understanding into population-level benefit. Where such programmes operate, most notably in the United Kingdom, Ireland, and the Nordic countries, DR has been displaced as the leading cause of certifiable blindness in the working-age population [7,8]. The shared architecture of these programmes is simple in principle: identify all people with diabetes through a registry, invite them at defined intervals for standardised retinal imaging, grade the images against a validated classification, and refer those with sight-threatening disease for timely ophthalmological management. In practice, building and sustaining this architecture demands reliable governance, a trained workforce, interoperable digital infrastructure, quality assurance (QA), and sustained political commitment.

Across much of Europe, systematic DR screening remains absent. Many countries rely on opportunistic case-finding within routine ophthalmological or diabetological care, an approach that consistently produces lower coverage and later-stage detection [9]. Croatia exemplifies this gap. With an estimated diabetes prevalence of 9–10% among adults, nearly 400,000 registered persons with diabetes, and a well-established national diabetes registry (CroDiab), Croatia possesses several prerequisites for systematic screening, yet no organised national programme has been established to date [10,11]. A draft national DR screening programme, anchored in CroDiab and the Central Health Information System of Croatia (CEZIH), is now under development.

Two developments, one organisational and one technological, have reshaped the options available to countries now building screening programmes. Telemedicine-based screening is well established and has been the dominant delivery model in European programmes launched over the past two decades [12]. In this model, non-mydriatic digital fundus photography is performed at primary-care or community sites and images are transmitted to a distant centre for grading. Artificial intelligence (AI), particularly deep learning–based image classification, has matured rapidly since 2016, with several systems now holding regulatory clearance for autonomous or assistive DR detection [13,14,15]. Together, these tools offer a viable route for resource-constrained health systems to achieve population-level screening without proportionally expanding their ophthalmology workforce.

This narrative review has three objectives. First, we summarise the global and European burden of DR and map established national screening programmes across Europe. Second, we examine the evidence base for telemedicine and AI-assisted DR screening, including regulatory considerations and real-world deployment data. Third, we apply these lessons to the Croatian context and outline a phased roadmap for implementing a national DR screening programme that integrates telemedicine and AI into the existing health system infrastructure.

## 2. Materials and Methods

This narrative review was based on a structured literature search. PubMed, Scopus, and Web of Science were searched from inception through January 2026 using combinations of the terms “diabetic retinopathy”, “screening”, “national programme”, “telemedicine”, “artificial intelligence”, “deep learning”, and “Europe”. The full Boolean search string combined controlled and free-text terms: (“diabetic retinopathy” OR “diabetic eye disease”) AND (“screening” OR “national programme” OR “telemedicine” OR “teleophthalmology” OR “artificial intelligence” OR “deep learning”) AND (“Europe” OR individual European country names). Reference lists of included studies and relevant guidelines from the International Council of Ophthalmology (ICO), the International Diabetes Federation (IDF), and the NHS Diabetic Eye Screening Programme were hand-searched. Eligible records were peer-reviewed original studies, systematic and narrative reviews, health technology assessments, and official national screening programmes or regulatory documents addressing DR epidemiology, screening organisation, telemedicine, AI-assisted grading, or programme economics; conference abstracts and commentaries without primary data were excluded, and only English- and Croatian-language sources were considered. The database search returned approximately 1500 records; after removal of duplicates and screening of titles and abstracts, around 170 full-text documents were assessed for eligibility, and 56 sources were included in the final synthesis. Grey literature, including national screening programme reports and health technology assessments, was included where peer-reviewed data were unavailable, because organised national screening programmes are frequently documented in government and agency reports rather than in the indexed literature. No formal quality appraisal or meta-analysis was performed. Croatian-specific data were obtained from the CroDiab national diabetes registry, the Croatian Institute of Public Health (HZJZ), and unpublished operational data from the pilot screening programme at KB Merkur, Zagreb. Throughout, Section 1, Section 2, Section 3, Section 4, Section 5 and Section 6 synthesise the published literature, whereas Section 7 (the Croatian context) and Section 8 (the proposed roadmap) present the authors’ own national-programme proposal, drawn from institutional pilot experience rather than from the reviewed literature; these latter sections should be read as a policy proposal rather than as evidence synthesis.

## 3. Burden of Diabetic Retinopathy and the European Screening Landscape

### 3.1. Epidemiological Burden

The International Diabetes Federation (IDF) estimates that 61 million adults in the European region live with diabetes, and a further 22 million have impaired glucose tolerance [1].

DR affects approximately one-third of people with diabetes, and roughly one-tenth have sight-threatening forms, namely proliferative DR (PDR) or DME [2,16]. A pooled analysis of 35 population-based studies reported a global prevalence of any DR of 34.6%, with PDR at 6.96% and DME at 6.81% [16]; more recent estimates project a rising burden through 2045 [2]. European prevalence data are broadly consistent with these figures, though regional variation exists, driven by differences in diabetes duration, glycemic control, screening ascertainment, and population demographics.

The economic burden is considerable. Vision loss from DR reduces labour productivity, increases disability payments, and generates large direct healthcare costs from vitreoretinal surgery, intravitreal injections, and long-term follow-up. Multiple cost-effectiveness analyses have concluded that systematic screening and early treatment are cost-saving relative to no screening, even under conservative assumptions about programme uptake and treatment efficacy [17,18,19].

### 3.2. Established National Programmes

The United Kingdom operates one of the world’s most extensively documented DR screening programmes. The NHS Diabetic Eye Screening Programme (DESP) in England, launched nationally in 2003, reached full coverage by 2008, and now invites all people with diabetes aged 12 years and over for annual two-field digital fundus photography [7,20]. Images are graded by trained, quality-assured non-medical graders against a standardised classification (the NHS DESP feature-based grading system, aligned with the International Clinical Diabetic Retinopathy Severity Scale). Approximately 2.7 million people attend screening in England alone each year, with uptake consistently above 80% [7,20]. Scotland, Wales, and Northern Ireland run parallel programmes with comparable structure.

A landmark analysis by Liew et al. demonstrated that DR is no longer the principal cause of certifiable blindness among working-age adults in England and Wales, a shift attributed to the screening programme [8]. In October 2023, England introduced risk-stratified recall, extending the interval to 24 months for individuals with two consecutive screens showing no DR. This change was supported by evidence that the annual yield of new referable disease in this low-risk group is extremely low [21].

Several factors explain why the UK programme has succeeded where others have stalled: central NHS funding removed the need to negotiate reimbursement for screening with insurers; a national diabetes register was already in place; and a dedicated non-medical grading workforce was created from scratch, avoiding reliance on scarce ophthalmologists. The programme also benefited from strong clinical leadership that maintained political support across multiple government cycles. These conditions are difficult to replicate in Bismarck-type insurance systems, where responsibility for preventive screening sits awkwardly between public health mandates and insurer-funded clinical care [22,23].

Ireland’s RetinaScreen programme, established in 2013, adopted a similar model with annual non-mydriatic photography, centralised digital grading, and population identification via the National Diabetes Register. Within its first years, the programme achieved uptake rates above 55% and identified substantial previously undiagnosed referable DR [24]. The programme has since expanded to cover over 200,000 eligible persons.

The Nordic countries have long been at the forefront of systematic DR screening. Denmark’s programme, operating since the 1990s, integrates screening into primary care and diabetology clinics, with ophthalmologist-supervised grading. Sweden uses a decentralised model in which county councils organise screening, often through non-mydriatic photography at primary care centres with images graded by ophthalmologists or trained nurses [25]. Iceland achieves near-universal coverage through a single national centre. Finland organizes screening at the municipal level, producing variable coverage but generally strong results in the larger urban centres [26].

The Netherlands integrates DR screening into its structured diabetes management programme (Diabetes Zorggroep model), with GPs coordinating annual retinal photography at practice or optometry sites. Portugal launched a national telemedicine-based screening programme in 2009–2011, using non-mydriatic cameras in primary care health centres and remote grading by ophthalmologists. The programme achieved broad geographic coverage despite a limited ophthalmology workforce in rural areas. Portugal’s experience is especially relevant for EU countries with limited specialist workforces: its programme demonstrated that telemedicine could deliver screening in a health system without a pre-existing non-medical grading workforce, though expansion beyond the initial regions proved slower than planned, partly because ophthalmologist-graders became a bottleneck as volume grew [27].

### 3.3. Countries with Partial or Absent Systematic Screening

Despite the strong evidence base, many EU member states lack formal national DR screening programmes. Germany, France, Italy, and Spain rely predominantly on opportunistic screening within ophthalmological or diabetological encounters, sometimes supported by regional or pilot initiatives. In Germany, the disease management programme for type 2 diabetes (DMP Type 2) recommends annual fundoscopy, but uptake data are incomplete, and the model relies on patient initiative and GP or diabetologist referrals. Italy has several regional telemedicine screening projects, especially in Tuscany and Veneto, but no unified national programme [9]. Spain has piloted telemedicine screening in the Basque Country, Catalonia, and the Canary Islands with promising results, yet national coordination is lacking.

Among newer EU member states, Slovenia has developed a structured screening programme through its primary care network, and Estonia has implemented telemedicine-based pilots. The broader picture in Central and Eastern Europe remains fragmented, with opportunistic screening remaining the norm. Croatia falls squarely in this category, possessing a strong registry infrastructure but lacking a systematic screening pathway. The barriers in these countries are informative: in the Czech Republic and Hungary, diabetes management guidelines recommend annual fundoscopy, but enforcement is weak, and no centralised grading or recall system exists. Poland, with over 2.5 million people with diabetes, has piloted telemedicine screening in several voivodeships but has not progressed to national coordination, in part due to fragmented governance among regional health funds [28]. Romania faces an acute ophthalmology workforce shortage in rural areas, making any specialist-dependent screening model impractical outside Bucharest and a handful of larger cities. The common thread across Central and Eastern Europe is not a lack of clinical awareness but the absence of the organisational infrastructure, dedicated funding, trained non-medical workforce, and political continuity required to convert guidelines into functioning population-level programmes (Table 1) [29,30].

### 3.4. Screening Models and Workforce

European programmes employ several organisational models. The dominant model in the UK and Ireland uses dedicated non-medical screener-graders who capture images in community or primary care settings and grade them at centralised reading centres. This model maximizes throughput and reserves ophthalmologist time for arbitration and clinical management. The Nordic model more frequently involves ophthalmologists or ophthalmic nurses in both imaging and grading, reflecting smaller populations and different workforce traditions. The Southern European approach, where it exists, tends to be ophthalmologist-centred, which limits scalability. Each model has trade-offs: the UK model requires a large, trained grading workforce and rigorous QA; the ophthalmologist-led model is capacity-constrained but may achieve higher per-grader accuracy.

Non-European comparators offer useful benchmarks. The United States Veterans Health Administration (VHA) operates one of the largest teleretinal screening programmes globally, using store-and-forward non-mydriatic photography at primary care clinics with remote grading by trained readers, and has substantially increased screening rates among enrolled veterans [32,33]. Singapore’s Integrated Diabetic Retinopathy Programme (SiDRP) screens the national diabetic population through a centralised telemedicine model and was among the first to integrate AI triage at scale [34].

## 4. Telemedicine and Technology in DR Screening

Telemedicine is now central to modern DR screening. The core model is store-and-forward digital fundus photography, captured at a remote site and graded asynchronously by a trained reader at a distant centre; this approach has accumulated a large evidence base over the past two decades [12,32]. Its advantages are clear: it decouples image acquisition from expert grading, extends screening coverage to areas with limited ophthalmology access, and creates a permanent digital record that can be reanalyzed, audited, and used for longitudinal monitoring.

### 4.1. Imaging Technology

Non-mydriatic digital fundus cameras are the standard imaging modality for population screening. Most programmes use one or two 45° macula-centred fields per eye; the English NHS DESP uses two-field imaging (macula-centred and disc-centred) [7]. This configuration provides adequate posterior pole coverage for detecting referable DR in most cases. The technical failure rate (images ungradable due to media opacity, poor fixation, or small pupils) typically ranges from 3% to 10% and is higher in elderly patients and those with cataracts [35,36]. Programmes manage this through mydriasis protocols for re-screening, slit-lamp biomicroscopy referral, or adjunctive optical coherence tomography (OCT) pathways. Since 2024, England has been rolling out OCT within its digital surveillance pathway, an advance that allows detection of DME at a subclinical stage [31,37].

Handheld and smartphone-based fundus cameras are now available as lower-cost alternatives suitable for mobile screening and resource-limited settings. Devices such as the Remidio Fundus on Phone and the Welch Allyn iExaminer have demonstrated acceptable image quality for DR grading when used by trained operators, though evidence for their use in large-scale population screening programmes is still maturing [38]. Ultra-widefield imaging is increasingly used in ophthalmological practice and some screening contexts, offering the ability to detect peripheral retinal pathology missed by standard two-field photography, but its role in population-level screening remains under evaluation due to cost and interpretation complexity [39].

### 4.2. Grading Standards and Quality Assurance

All mature screening programmes use a structured grading classification aligned with or derived from the ETDRS severity scale. The English NHS DESP employs a feature-based grading system (R0–R3 for retinopathy, M0–M1 for maculopathy) that maps onto the International Clinical Diabetic Retinopathy Severity Scale. Scotland uses a similar system. Ireland’s RetinaScreen uses the International Clinical Diabetic Retinopathy Severity Scale [7,24]. The grading process is tiered: primary grading by trained non-medical graders, secondary grading for positive or uncertain cases, and arbitration by ophthalmologists for disagreements or complex findings. This tiered system is integral to quality control.

QA encompasses internal consistency checks (test-and-training sets, intergrader agreement monitoring), external quality assurance visits, fail-safe processes to ensure no patient is lost to follow-up, and regular clinical governance reviews. The English programme publishes quarterly key performance indicators (KPIs), including uptake rates, technical failure rates, grading turnaround times, and referral-positive rates [7,40]. These KPIs serve both as quality benchmarks and as epidemiological surveillance tools.

## 5. AI-Assisted DR Screening

### 5.1. Validation Evidence

The application of deep learning to retinal image analysis is one of the more extensively studied clinical applications of AI; however, most evidence comes from diagnostic-accuracy studies rather than trials measuring patient outcomes. In 2016, Gulshan and colleagues at Google published a study in JAMA demonstrating that a deep convolutional neural network, trained on over 128,000 retinal images graded by a panel of ophthalmologists, could detect referable DR with a sensitivity exceeding 96% and specificity above 93% across two independent validation sets [13]. These figures approached or exceeded the performance of individual ophthalmologists. Ting et al. subsequently validated a deep learning system on nearly 72,000 images from the Singapore national screening programme and 10 multi-ethnic cohorts, reporting an area under the receiver operating characteristic curve (AUC) of 0.936 for referable DR and 0.958 for vision-threatening DR [14].

The IDx-DR system (now LumineticsCore, Digital Diagnostics, Coralville, IA, USA) was the first AI device to receive United States Food and Drug Administration (FDA) De Novo clearance for autonomous detection of DR in April 2018. In its pivotal trial of 900 participants across 10 primary-care sites, the system achieved a sensitivity of 87.2% and a specificity of 90.7% for detecting more-than-mild DR [15]. EyeArt (Eyenuk, Los Angeles, CA, USA) received FDA clearance in 2020 and reported sensitivities above 95% for referable DR in multi-centre validation [41]. In Europe, both systems have obtained CE marking under the Medical Devices Regulation, enabling deployment in EU member states.

A systematic review and meta-analysis by Islam et al., pooling data from deep learning studies for DR detection, reported a summary sensitivity of 80–97% and specificity of 88–99%, depending on the referral threshold, dataset, and whether autonomous or assistive deployment was modelled [42].

Real-world performance data from deployed systems in Thailand, India, and several European pilot sites have generally confirmed high sensitivity for referable DR, though specificity in operational settings tends to be lower than in validation studies, a pattern attributable to differences in image quality, patient demographics, and disease spectrum [43,44].

### 5.2. Deployment Models

AI can be integrated into DR screening pathways at several points. In the autonomous model, the AI system provides a binary screen-positive or screen-negative result without human grading of negative images. The FDA-cleared IDx-DR system operates in this mode. In the assistive or triage model, AI pre-screens all images and identifies those likely to be positive, which are then reviewed by human graders; images classified as clearly negative may bypass human grading entirely or undergo reduced-level review. This approach is increasingly favoured in European programmes because it preserves a human-in-the-loop safeguard while substantially reducing grading workload [45].

A third model uses AI for quality control and safety-netting: detecting ungradable images in real time, flagging discordances between AI and human grading, and identifying patients whose images suggest pathology not captured by the primary grading classification (e.g., glaucoma suspects, age-related macular degeneration). Scotland’s screening programme has piloted AI in a pre-screening triage role, and early data suggest it can reduce the human grading burden by 40–50% without compromising sensitivity for referable disease [31,46].

### 5.3. Limitations and Regulatory Considerations

Several unresolved challenges constrain large-scale AI deployment. Dataset bias remains a serious concern: most deep learning systems have been trained predominantly on images from South and East Asian or North American populations, and performance may differ when these systems are applied to European populations with different pigmentation and comorbidity profiles, or to images acquired with different equipment [29]. Local validation is therefore essential before any system is deployed operationally in a new setting. Regulatory frameworks in the EU under the Medical Devices Regulation (MDR 2017/745) and, prospectively, the AI Act, impose rigorous requirements for clinical evidence, post-market surveillance, and transparency that AI developers and health systems must meet [47,48]. Importantly, most commercially available systems have been validated only in their settings of origin and lack local validation, regulatory approval, and post-deployment performance monitoring in European screening populations. These are prerequisites for, not consequences of, deployment.

Explainability, the ability to provide clinicians and patients with understandable reasons for an AI decision, is both a clinical governance requirement and an ethical obligation increasingly emphasised in medical AI policy. Current deep learning systems remain largely opaque in their decision-making, though heatmap-based visualizations (such as Grad-CAM) provide partial interpretability [49]. Liability in the event of a missed diagnosis or false negative is another unresolved issue, with no settled medico-legal framework across EU jurisdictions.

Prospective, randomized evidence comparing AI-assisted screening with standard care on patient-relevant outcomes (visual acuity preservation and rate of progression to advanced disease) remains largely absent. The available evidence is predominantly diagnostic accuracy studies, many of which are retrospective. Although the sensitivity and specificity figures are reassuring, they do not directly demonstrate that AI-based screening programmes produce better patient outcomes than those using human grading alone. Early prospective evidence is emerging: a randomized trial of autonomous AI screening in youth with diabetes reported substantially improved screening and follow-up completion [50], although trials powered for visual-outcome endpoints are still pending.

## 6. Barriers and Facilitators to DR Screening Implementation

The gap between evidence and implementation in DR screening is substantial. Countries considering new programmes face interrelated challenges at the health system, workforce, economic, patient, and regulatory levels.

At the health-system level, successful programmes require a functioning diabetes registry with high population coverage, interoperable electronic health records, reliable referral pathways to ophthalmology services, and sustained political commitment across election cycles. Countries with Bismarck-type social insurance systems, such as Croatia, Germany, and the Netherlands, face specific challenges regarding contracting and reimbursement: who pays for screening, how it is coded, and how insurance funds interact with public health mandates. In Beveridge-type systems (the UK, the Nordic countries), central funding and commissioning are simpler, though local variation in implementation quality persists [51].

Workforce limitations are a universal barrier. Training a cadre of non-medical graders, as in the UK model, takes 12–18 months and requires ongoing QA investment. Ophthalmologist-led grading, while requiring less training infrastructure, diverts specialist time from surgical and clinical care. AI can partially address this bottleneck, but its deployment creates new training needs: technicians to operate cameras and manage imaging workflows, informaticians to maintain AI systems, and clinical leads with the competence to oversee AI-human hybrid grading.

Patient-level barriers include lack of awareness that diabetes can cause blindness, fear of diagnosis, travel distance to screening sites, and competing health priorities. Uptake is consistently lower among socioeconomically deprived groups, ethnic minorities, younger adults with type 1 diabetes, and men [52,53]. Mobile screening units, evening and weekend appointments, community-based sites, and culturally tailored invitation materials have all been shown to improve attendance in underserved populations.

Patient engagement deserves more attention than it typically receives in programme design. The UK experience shows that even a well-resourced programme plateaus at 80–83% uptake [46], with the remaining 17–20% disproportionately concentrated among younger men, people with type 1 diabetes, and socially deprived groups. Passive invitation letters are insufficient for these populations. Strategies shown to improve uptake include GP endorsement at routine diabetes consultations, peer-led community outreach, integration of screening with other diabetes care appointments, and personalized digital reminders via SMS or patient portals [54]. For countries in the design phase, building these engagement strategies into the programme from the outset, rather than retrofitting them after coverage stalls, is likely to be both more effective and less costly.

Economic modelling supports the cost-effectiveness of systematic DR screening across a variety of health-system settings, but start-up costs are non-trivial: capital investment in cameras and IT infrastructure, recurrent workforce costs, and ongoing QA. For EU countries with constrained health budgets, securing dedicated programme funding, as opposed to embedding screening costs within general ophthalmology budgets, is a political and administrative challenge [17].

## 7. The Croatian Context

Croatia is a smaller EU member state (population approximately 3.9 million) with a Bismarck-type health insurance system administered by the Croatian Health Insurance Fund (HZZO). Healthcare delivery is structured through a network of primary care practices (family medicine), county-level general hospitals, and university hospital centres in Zagreb, Split, Rijeka, and Osijek. Ophthalmology services are concentrated in secondary and tertiary centres; at present, 15 centres across the country possess the full complement of equipment required for DR diagnosis and treatment (OCT, fluorescein angiography, panretinal photocoagulation laser, and intravitreal injection capability), while a further four hospitals have OCT devices but lack complete therapeutic infrastructure.

The prevalence of diabetes in Croatia is estimated at 9–10% of the adult population. According to the most recent CroDiab report, 396,005 persons with diagnosed diabetes were registered in 2024, with around 40,000 new diagnoses recorded annually, underscoring the scale of the diabetes epidemic in Croatia [10]. An estimated 28.5% of adults aged 20–79 years with diabetes remain undiagnosed [1], placing the true burden above 500,000 persons. Diabetes is now the third leading cause of death in Croatia, accounting for 7.7% of all deaths in 2023 [10]. Prevalence rises sharply after age 40, reaching about 28% among persons older than 70 years. CroDiab, established in 2000 and mandatory since 2004, is a web-based registry maintained by the Croatian Institute of Public Health (HZJZ) that collects clinical and laboratory data from primary and secondary care on all patients diagnosed with diabetes. The registry is linked to CEZIH, Croatia’s central electronic health information system, enabling data sharing across healthcare levels. This infrastructure, a population-based diabetes registry integrated with electronic health records, is a major asset for screening programme design, as it provides the denominator population and a mechanism for invitation and recall.

At present, DR detection in Croatia occurs opportunistically. Patients may be referred by their GP or diabetologist to an ophthalmologist for fundus examination, but there is no systematic invitation, no standardised imaging protocol for screening, and no population-level coverage data. Clinical experience suggests that a large proportion of people with diabetes have never had a fundus examination, and that many present to ophthalmology services with advanced DR or DME. The referral pathway is fragmented: there is no dedicated grading workforce, no centralised reading centre, and no structured QA for DR detection.

However, a local screening programme has been operating for over two and a half years at the Department of Diabetic Eye Complications, University Clinic Vuk Vrhovac, KB Merkur. This pilot has demonstrated a DR prevalence of 40% among screened patients with type 2 diabetes, with higher blood pressure and infrequent prior fundus examinations identified as significant risk factors for sight-threatening disease [11]. If this prevalence, likely an overestimate given the clinic-based sample, is cautiously applied to the national population, more than 130,000 persons in Croatia may harbour some form of retinopathy, the majority without symptoms. Of these, roughly 15% may have moderate-to-severe non-proliferative DR, around 5% proliferative disease, and around 7% clinically significant diabetic macular edema. These figures, even allowing for overestimation, highlight the urgency of transitioning from opportunistic case-finding to organised population-level screening. For context, population-based studies report any-DR prevalence of approximately 34.6% [16], so the clinic-derived 40% figure should be interpreted as an upper bound rather than a population estimate.

We developed a draft national DR screening programme at the Department of Diabetic Eye Complications, University Clinic Vuk Vrhovac, Merkur University Hospital (Klinika za dijabetes, endokrinologiju i bolesti metabolizma Vuk Vrhovac, KB Merkur), building on the knowledge, methodology, and operational experience accumulated over two and a half years of pilot screening at that institution. The programme document has been submitted to the Ministry of Health and is under review; formal adoption is pending. The programme is designed as a decentralised, telemedicine-based system anchored in the CroDiab registry as the population identification mechanism. It proposes non-mydriatic digital fundus photography at primary care sites, community health centres, hospitals, and mobile screening units, with centralised grading by trained ophthalmologists at dedicated reading centres. The digital architecture integrates with CEZIH, eNaručivanje (the national e-referral and scheduling system), and eKarton (the electronic patient record), enabling automated patient identification, invitation, appointment scheduling, image transfer, grading, result communication, and referral generation. Patients with sight-threatening findings would be referred via automatically generated e-referrals to one of the existing treatment centres. The programme envisions staged implementation across three phases, from a pilot in two to three counties through to full national coverage targeting 400,000 screenings per year, with a set of KPIs modelled on European best practice. The proposed pathway also reflects several clinical considerations relevant to screening generally: screening intervals are differentiated by diabetes type, with type 1 patients entering screening five years after diagnosis (and not before age 12) and type 2 patients from diagnosis, reflecting the differing natural history of retinopathy; an ungradable image (most often caused by cataract or other media opacity) is treated as a referral trigger requiring mydriatic re-imaging and, if still ungradable, slit-lamp examination, rather than as a normal result; and maculopathy (M1) findings are routed to optical coherence tomography for confirmation of diabetic macular edema before treatment referral.

## 8. A Proposed Roadmap for National DR Screening in Croatia

**Figure 1:**
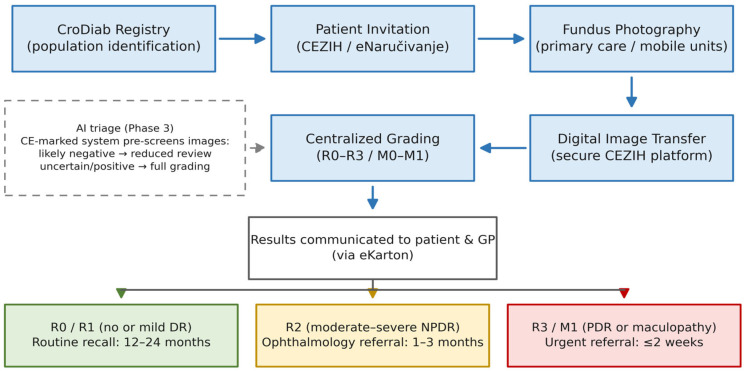
Proposed screening workflow for the Croatian national DR screening programme. The final row summarizes patient management based on diabetic retinopathy grading. Green indicates no or mild diabetic retinopathy (R0/R1), requiring routine recall in 12–24 months. Yellow represents moderate-to-severe non-proliferative diabetic retinopathy (R2), requiring ophthalmology referral within 1–3 months. Red denotes proliferative diabetic retinopathy or diabetic maculopathy (R3/M1), requiring urgent ophthalmology referral within ≤2 weeks.

Drawing on the European evidence reviewed above, the specific characteristics of the Croatian health system, and the operational experience of the KB Merkur pilot, we outline a phased implementation roadmap. The model is designed to be scalable and regionally adaptable, preserving uniform standards of care while accommodating differences in local capacity. It builds on the existing CroDiab registry and CEZIH digital infrastructure, incorporates telemedicine as the default delivery mechanism, and leaves a defined pathway for future AI integration (Figure 1).

The workflow shows patient identification via the CroDiab registry, telemedicine-based image acquisition, centralised grading with referral pathways stratified by disease severity, and planned AI triage integration (dashed box, Phase 3). CEZIH, Central Health Information System of Croatia; GP, general practitioner; NPDR, non-proliferative diabetic retinopathy; PDR, proliferative diabetic retinopathy; R0–R3/M0–M1, retinopathy and maculopathy grading scales. This figure depicts a proposed workflow that has not yet been operationally validated at the national scale.

### 8.1. Phase 0–1: Planning, Foundation Building, and Pilot (Months 0–18)

**Figure 2:**
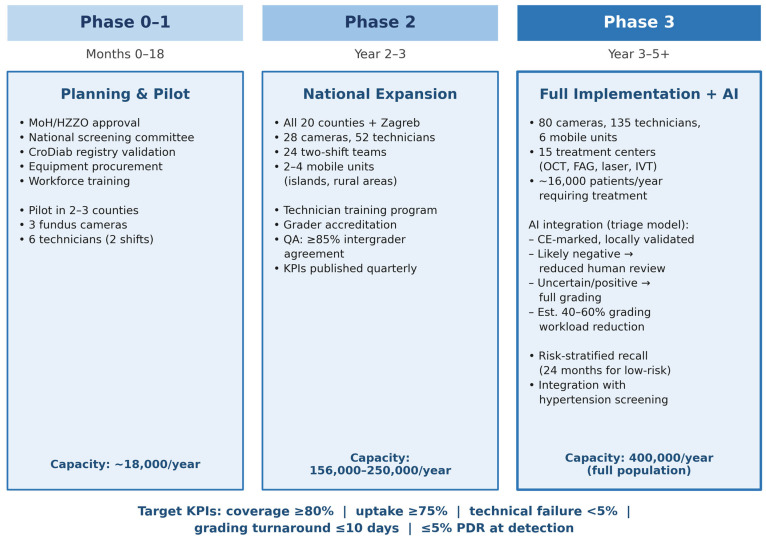
Phased implementation timeline for the Croatian national DR screening programme, showing resource allocation, capacity targets, and key performance indicators. FAG, fluorescein angiography; IVT, intravitreal therapy; OCT, optical coherence tomography; PDR, proliferative diabetic retinopathy. The phases, resources, and targets shown represent a proposed plan rather than a validated operational pathway.

The initial six months (Phase 0) should be devoted to finalizing the programme document, obtaining formal approval from the Ministry of Health, HZJZ, and HZZO, and establishing the governance framework (Figure 2). A dedicated national screening committee, including representation from ophthalmology (the Croatian Ophthalmological and Optometric Society, HOOD), diabetology, primary care, public health, patient organisations, and health informatics, should be constituted with a defined mandate and budget line. National and regional coordination teams should be appointed, and clinical protocols, technical standards, and imaging specifications should be codified. Equipment procurement (fundus cameras, IT infrastructure, mobile units) and initial workforce training should proceed in parallel.

The CroDiab registry should be validated as the population denominator. The completeness of registration, the accuracy of contact details, and the linkage to CEZIH must be audited. The target population includes all persons with type 1 and type 2 diabetes aged 12 and over, with screening intervals aligned to the International Clinical Diabetic Retinopathy Severity Scale: persons with type 1 diabetes should enter screening five years after diagnosis (and not before age 12), whereas those with type 2 diabetes should be screened at or shortly after diagnosis. Invitation and recall should be managed centrally through CEZIH and eNaručivanje, with automated scheduling and fail-safe tracking. Priority groups should be flagged for expedited inclusion: persons with diabetes duration exceeding ten years who have never had a fundus examination, those with chronically poor glycemic control (HbA1c > 8.0%), pregnant women with pre-gestational diabetes, and socially vulnerable or hard-to-reach populations. These priority groups should be screened on an expedited basis and reviewed at least annually rather than on the standard 12–24-month recall. Pregnant women with pre-gestational diabetes require a distinct, intensified schedule, reflecting the accelerated progression of DR in pregnancy: retinal assessment at pregnancy booking (or pre-conception where feasible) and at least once in each trimester, with more frequent review if any retinopathy is present.

Pilot screening (Phase 1, months 6–18) should begin in two to three counties selected for geographic and demographic diversity, replicating the operational model already validated at KB Merkur. The pilot configuration envisions three fundus cameras and six trained technicians working in two shifts, yielding an annual capacity of around 18,000 patients. These capacity figures derive from operational norms validated over two and a half years at the KB Merkur pilot. A single camera staffed by two technicians across two shifts processes approximately 30 patients per day; over 220 working days per year, and after applying a 90% appointment-efficiency factor that accounts for non-attendance, staff sick leave, and equipment downtime, this corresponds to roughly 6000 completed screening episodes per camera-year (a single-shift, single-technician configuration yields approximately 3000). Three cameras therefore yield around 18,000 episodes annually. Applying the same norm, the Phase 2 configuration of 28 cameras (52 technicians in 24 two-shift teams, plus two to four mobile units) yields approximately 156,000 episodes per year, and the Phase 3 configuration of 80 cameras (135 technicians in 55 two-shift and 19 single-shift teams, plus six mobile units) yields approximately 400,000 episodes per year, sufficient to cover the registered diabetic population. Programme key performance indicators cap the image-repeat rate at below 10% and the ungradable-image rate at below 5%. On the reading side, graders require an average of 3.2 min per image set (2 min for a negative and 5 min for a positive result), equivalent to approximately 18 reads per hour or 144 per eight-hour day; at full national volume this corresponds to the equivalent of, for example, 20 ophthalmologist-graders each contributing approximately 22 h per week, a workload that AI-assisted triage is intended to relieve. Each pilot site should be equipped with at least one non-mydriatic digital fundus camera (minimum 45° field of view, ≥6-megapixel resolution, autofocus capability), operated by trained medical technicians or nurses at primary-care practices, diabetes outpatient clinics, or community health centres. The imaging protocol, following the standards set by Aldington et al. [55], requires a minimum of two fields per eye (macula-centred and nasal periphery) for patients aged 35 and over, and three fields per eye (adding temporal periphery) for younger patients. Mydriasis should be avoided for routine screening; where image quality is inadequate, tropicamide 0.5–1.0% may be applied and imaging repeated. Images failing to meet quality criteria on repeat imaging should prompt a direct ophthalmological referral.

Grading during the pilot phase should be performed by ophthalmologists with additional training in DR image interpretation, operating within centralised reading (grading) centres using a secure web-based platform integrated with CEZIH. The grading classification should align with the modified International Clinical Diabetic Retinopathy and Diabetic Macular Edema Severity Scales, using the R0–R3/M0–M1 notation adapted from the NHS DESP model. Referral thresholds should be set as follows: R0/M0 (no DR), routine recall at 12–24 months; R1 (mild NPDR), recall at 12 months; R2 (moderate-to-severe NPDR), ophthalmological referral within one to three months; R3 (proliferative DR), urgent referral within two weeks; M1 (clinically significant maculopathy), referral for OCT and treatment. An automated alert system should be built into the digital platform to ensure that R3 and M1 findings trigger immediate notification to the nearest treatment centre. Reading centres should return grading results within 10 working days of image receipt, and 10–15% of all readings should undergo double grading for quality assurance. This double-grading sample is additional to the mandatory secondary grading of all positive and ungradable images and follows the tiered quality-assurance practice of established programmes rather than a single fixed published standard [7]. During the pilot phase, the grading workforce will consist of ophthalmologists; as the programme matures, a pathway for trained non-medical graders may be explored in line with international models.

### 8.2. Phase 2: National Coverage Expansion (Years 2–3)

Based on pilot results, Phase 2 should extend screening to all 20 counties and the City of Zagreb (Figure 2). The proposed configuration for this phase envisions 28 fundus cameras and 52 technicians organised into 24 two-shift teams, supplemented by two to four mobile screening units for islands and sparsely populated rural areas. This configuration yields an estimated annual capacity of 156,000 screened patients, rising to 250,000 when repeat screenings begin in the third year. At least one camera should be deployed per county, with additional units in high-population counties (City of Zagreb, Zagreb County, Split-Dalmatia, Primorje–Gorski Kotar, and Osijek-Baranja). Mobile units equipped with a fundus camera, a laptop running electronic medical record software, and internet connectivity are essential for reaching island and rural communities that would otherwise face prohibitive travel distances. Education programmes should be expanded, with structured training for technicians (a two-day online module plus a practical workshop covering fundus camera operation, image quality criteria, patient management, and data handling) and for ophthalmologist-graders (a two-day theoretical programme plus a three-day practicum covering classification systems, standardised grading, digital platform use, and referral protocols). Accreditation standards should be developed, with re-certification every three to five years, and a national register of certified programme personnel should be maintained.

QA processes must be embedded from the outset and intensified during this scaling phase. Internal QA should include quarterly distribution of test-and-training image sets, intergrader agreement monitoring (assessed by Cohen’s or weighted κ, with a target of κ ≥ 0.8, since raw percent agreement overstates concordance when negative cases predominate in screening), and technical image quality audits (target ≥ 90% gradable images, <10% repeat rate). External QA should encompass an annual clinical governance review, a comparison of programme detection rates with expected epidemiological prevalence, and the regular publication of KPIs. Key performance indicators should include, at minimum, coverage (target ≥ 80% of the eligible population within five years), uptake (target ≥ 75% of invited persons), technical failure rate (<5%), grading turnaround time (≥90% of readings completed within 10 working days), referral-positive rate, and sight-threatening DR detection rate (target: ≤5% proliferative disease among newly detected cases, reflecting the goal of catching disease at early stages). Communication of results to patients and their treating physicians should be automated through CEZIH and eKarton, with urgent findings (R3, M1) triggering direct notification to the nearest treatment centre and automatic generation of a specialist e-referral.

### 8.3. Phase 3: Full Implementation and AI Integration (Years 3–5+)

After two to three years of operation, the programme should reach full operational capacity through additional equipment procurement and workforce expansion (Figure 2). The target configuration for Phase 3 envisions 80 fundus cameras and 135 technicians (organised into 55 full two-shift teams and 19 single-shift teams), supplemented by six mobile screening units, yielding an annual capacity of 400,000 screened patients, sufficient to cover the entire registered diabetic population. The existing network of 15 treatment centres (five university hospital centres and ten county or clinical hospitals with full OCT, FAG, laser, and intravitreal injection capability) is expected to be adequate across all three phases, with further capacity available by upgrading the hospitals that currently possess OCT but lack complete therapeutic equipment. Based on the expected 40% prevalence of DR and the roughly 10% rate of advanced disease requiring treatment, the programme would generate an estimated 16,000 patients per year requiring ophthalmological intervention, corresponding to a mean daily intake of approximately five new patients per centre across 15 centres and 220 working days. IT system optimisation, workflow automation, and integration with other preventive programmes (e.g., hypertension screening) should be pursued during this phase to maximise operational efficiency.

AI should be introduced in a controlled, stepwise manner during this phase. We recommend beginning with an assistive (triage) model rather than autonomous deployment, given the regulatory and clinical governance considerations discussed above. In this model, all images would be processed by a CE-marked AI system, which would classify them as likely negative (no referable DR), uncertain, or likely positive. Images classified as likely negative by AI would undergo a reduced-level human review (e.g., a single primary grader rather than full tiered grading); images deemed uncertain or positive would proceed through full human grading. A dedicated image-quality output is retained as a fourth, distinct category: ungradable images are never classified as likely negative but instead trigger automatic re-imaging (with mydriasis where indicated) and, if still ungradable, human grading or direct ophthalmology referral. This approach can reduce the grading workload by an estimated 40–60% while maintaining the safety net of human oversight [31,45].

Before deployment, any AI system used in the Croatian programme must undergo local validation on a representative dataset of Croatian fundus images that captures the full spectrum of disease severity, image quality, and camera types used in the programme. This validation should be conducted as a prospective diagnostic accuracy study with predefined performance thresholds (e.g., sensitivity ≥ 90% and specificity ≥ 80% for referable DR), consistent with the performance standards set by the English programme for human graders. The AI system must hold a valid CE marking under the EU MDR, and compliance with the evolving EU AI Act requirements should be ensured [47].

Over time, as confidence in AI performance accumulates and the regulatory environment matures, a shift toward greater AI autonomy may be considered, with AI providing definitive screen-negative results for clearly normal images. Such a transition should be data-driven, guided by an ongoing audit of AI–human concordance and safety monitoring.

### 8.4. Governance and Sustainability

Long-term sustainability depends on dedicated, ring-fenced programme funding. Screening should be classified as a preventive health service under the HZZO benefit package, with a defined reimbursement tariff for image acquisition, grading, and programme management. Financial modelling based on the draft programme document estimates total five-year costs at EUR 20.6 million across all three phases, with the majority (roughly 85%) attributable to human-resource costs, a pattern consistent with other population-based screening programmes. Start-up investments in equipment and training are concentrated in years one and three (corresponding to the Phase 1/2 and Phase 3 transitions), after which the programme enters a phase of stable annual operating costs of around EUR 5 million. These costs must be weighed against the projected savings: scenario-based modelling suggests that 2000 to 3000 cases of blindness could be prevented over five years, generating direct healthcare savings of EUR 14–30 million from avoided vitreoretinal surgery, intravitreal injections, and long-term follow-up, with additional societal savings of EUR 5–10 million from reduced disability payments and preserved labour productivity. Diversified funding sources, including the state budget, HZZO, EU structural and health programme funds (e.g., INTERREG), and carefully regulated industry partnerships, should be pursued to ensure financial resilience. These estimates should be interpreted with caution pending independent health-economic evaluation; a formal cost-effectiveness analysis is planned as a companion study. The cost and savings figures above are therefore indicative planning estimates, not cost-effectiveness results. The projections assume screening uptake of ≥70%, treatment compliance consistent with existing Croatian ophthalmology referral patterns, and cost inputs derived from HZZO reimbursement tariffs current as of 2024.

Data governance must ensure compliance with the EU General Data Protection Regulation (GDPR) [56] while enabling the data flows necessary for screening: invitation management, image storage, grading records, referral tracking, and outcome monitoring. The CEZIH infrastructure, if appropriately adapted, can support these requirements. A national screening database, linked to CroDiab and the hospital information systems, should serve as the authoritative data source for programme management and evaluation.

## 9. Discussion and Future Directions

This review supports three main conclusions. Systematic DR screening programmes, when properly implemented, reduce the burden of diabetes-related blindness. The UK experience provides the strongest proof of concept, but programmes across different European health systems, from the centralised Nordic models to the decentralised Dutch and Portuguese approaches, demonstrate that the core principles are adaptable. Telemedicine is now the standard delivery mechanism, and AI has reached diagnostic-accuracy levels in validation studies that may support cautious, staged integration into screening pathways, provided it is introduced with appropriate clinical governance, local validation, and regulatory compliance. The transferability of the UK experience should nonetheless be interpreted cautiously: the NHS is a tax-funded, single-payer system with a centrally commissioned national screening programme and an established non-medical grading workforce, whereas Croatia’s Bismarck-type, insurance-based system has neither a single commissioning authority for screening nor a dedicated grading workforce, so the organisational preconditions that enabled the English programme cannot be assumed to transfer directly.

A screening programme can deliver benefit only if downstream treatment services can absorb the referrals it generates. Croatia currently has 15 centres equipped for full DR management (OCT, fluorescein angiography, panretinal photocoagulation, and intravitreal therapy), with a further four hospitals holding OCT capacity that could be upgraded. Given the projected case mix (approximately 40% of screened patients with some DR, of whom about 10% require intervention), full national screening of 400,000 people per year would generate on the order of 16,000 patients per year requiring treatment, equivalent to roughly five new patients per working day distributed across 15 treatment centres. This volume appears manageable within the existing network, provided that laser and intravitreal-injection capacity is expanded incrementally in step with screening rollout and that the four OCT-only sites are upgraded during Phase 3. The principal constraint, however, is the grading workforce: Croatia has no dedicated non-medical grading workforce, so the programme initially depends on redeploying ophthalmologist time for grading until grader training and AI-assisted triage relieve this bottleneck. Aligning the pace of screening expansion with treatment and grading capacity, rather than outpacing it, is therefore a core design principle of the proposed roadmap.

Croatia’s position is, in some respects, advantageous. The country is building a programme from scratch, which means it can adopt current best practice without the legacy constraints that older programmes face. The CroDiab registry and CEZIH provide a digital backbone that many countries implementing screening in the 2000s had to build from the ground up. A local pilot programme at KB Merkur has already generated two and a half years of operational data, validated workflows, and documented a DR prevalence of 40%, providing an evidence base that few countries have at the outset of national programme design. The registered diabetic population of approximately 400,000 persons (and growing by 40,000 per year) makes a telemedicine-based model with centralised grading operationally feasible; this is a very different proposition from scaling such a model in a country of 50 or 80 million.

At the same time, constraints are real. The ophthalmology workforce is limited, and training a non-medical grading workforce will take time. The health system is accustomed to specialist-centred care, and shifting DR detection to a primary-care–based, non-physician–led screening model represents a cultural as well as an organisational change. Securing sustained political commitment and dedicated funding in a health system with competing priorities, including an ageing population, brain drain among health professionals, and post-pandemic recovery, will require persistent advocacy from the ophthalmology, diabetology, and public-health communities. We acknowledge that the roadmap proposed here originates from the authors’ institutional work at KB Merkur; the review framework is intended to position this proposal within the broader European evidence base and invite external scrutiny, not to substitute for independent evaluation.

The AI question is especially pertinent for Croatia. For a country with a limited grading workforce, AI-assisted triage is not merely a technological enhancement but potentially a prerequisite for achieving population-level screening coverage within a realistic timeframe. The assistive model we recommend is conservative: it does not replace human judgment but extends it. As the evidence base and regulatory clarity improve, Croatia could be among the first European countries to implement a fully integrated telemedicine–AI screening model at a national scale, a prospect of direct relevance to other smaller EU member states facing similar challenges, particularly those in Central and Eastern Europe with comparable population sizes, Bismarck-type insurance systems, and limited ophthalmology workforces.

Several research priorities deserve mention. Prospective trials comparing AI-assisted with human-only grading on patient outcomes are urgently needed. The Croatian programme, if implemented, offers a natural setting for such a trial. A cluster-randomized design in which counties are allocated to AI-assisted versus human-only grading pathways, with sight-threatening DR detection rates and time-to-treatment as co-primary endpoints, would be feasible within the phased rollout structure and could generate evidence directly relevant to other European countries considering similar programmes. Health-economic evaluations of AI integration in European settings, using country-specific cost and epidemiological data, would strengthen the business case. Studies on patient and provider acceptability of AI in DR screening, particularly in Central and Eastern European contexts, remain scarce. Finally, developing datasets representative of European populations, including underrepresented groups, is critical to ensuring equitable AI performance.

Several limitations should be acknowledged. As a narrative review, the literature search was not systematic, and selection bias in the included studies cannot be ruled out. The Croatian prevalence estimate of 40% DR among screened patients derives from a single-centre clinic-based sample at KB Merkur, which may overestimate population prevalence due to referral bias. Cost projections are based on unpublished modelling and have not undergone independent economic evaluation. The phased roadmap, while grounded in European best practice, has not been prospectively validated, and implementation feasibility will depend on political, financial, and workforce factors that are difficult to predict. Finally, the AI deployment recommendations reflect the current regulatory and evidence environment, which is evolving rapidly; the specific systems and thresholds recommended may require revision as new data and regulatory frameworks emerge.

## 10. Conclusions

The evidence from established European programmes is consistent: systematic DR screening, built on telemedicine and supported by trained grading workforces and rigorous QA, reduces diabetes-related blindness. Croatia possesses the essential prerequisites (a near-complete diabetes registry, a functioning health information system, and validated pilot data) but lacks the organised screening pathway to connect them. The phased roadmap outlined here offers a practical route from the current opportunistic model to population-level coverage, with AI-assisted triage as a means of achieving sustainable grading capacity. The roadmap proposed here is intended as a reasonable, evidence-informed basis for national planning rather than a prediction of outcomes. Implementation will require dedicated funding, sustained political commitment, and investment in a non-medical screening workforce. If these conditions are met, Croatia could plausibly reduce the burden of advanced DR over the coming decade and generate operational evidence of value to other European countries at a similar stage of programme development [57].

## Figures and Tables

**Table 1:** Comparison of established and planned DR screening programmes in Europe. CEE, Central and Eastern Europe; DR, diabetic retinopathy; AI, artificial intelligence. Uptake/coverage figures are approximate and reflect the most recent available data. “Variable” indicates municipal or regional variation in coverage. Coverage bands: ≥80% = established high uptake; 50–79% = moderate; Near-universal = single national centre with near-complete coverage; Developing/Planned = not yet at national scale. Figures are approximate and reflect the most recent available national sources, cited per row.

Country	Year Launched	Screening Model	Uptake/Coverage	Key Outcome
England	2003	Non-medical graders, centralised reading centres, 2-field photography	≥80% [7,20]	DR no longer leading cause of working-age blindness
Scotland	2003	Parallel to England; AI triage piloted	≥80% [31]	40–50% grading workload reduction with AI
Ireland	2013	Centralised digital grading via RetinaScreen	50–79% (rising) [24]	Substantial undiagnosed referable DR identified
Denmark	1990s	Integrated into primary care/diabetology clinics	Variable (regionally high) [9,25]	Long-standing reductions in DR-related visual loss
Sweden	1990s	Decentralised, county-organised, nurse/ophthalmologist grading	Variable (regionally high) [25]	Reduced new blindness by >30% (Stockholm data)
Iceland	–	Single national centre	Near-universal [25]	Near-complete population coverage
Finland	Variable	Municipal-level organisation	Variable [26]	Strong in urban centres
Netherlands	Integrated	GP-coordinated, Diabetes Zorggroep model	Variable (high) [9]	Embedded in structured diabetes management
Portugal	2009–2011	Telemedicine, non-mydriatic cameras in primary care	Variable (broad) [27]	Demonstrated feasibility without non-medical graders
Slovenia	Structured	Primary care network–based	Developing (partial) [30]	Most advanced CEE programme
Croatia	Planned	Proposed telemedicine + AI triage via CroDiab/CEZIH	Planned (target ≥ 80%) [10,11]	Pilot at KB Merkur: 40% DR prevalence

## Data Availability

This narrative review synthesises previously published data, which are available in the cited sources. Aggregate operational data from the KB Merkur pilot screening programme and the financial-modelling inputs underlying the cost projections are not publicly available, as they form part of an ongoing programme-development process, but are available from the corresponding author on reasonable request, subject to institutional approval.
